# Supplementary material for: The interactome of intact mitochondria by cross-linking mass spectrometry provides evidence for coexisting respiratory supercomplexes
Source: Mol Cell Proteomics. 2017 Dec 8;17(2):216–32. doi: 10.1074/mcp.RA117.000470 (PMC5795388; doi:10.1074/mcp.RA117.000470)
Supplement: Supplemental Data [file supp_RA117.000470_133922_0_supp_23978_fzffwf.zip › spectra_annotation/mito_DR_spectra_annotation/329-1-10-1-8-1.pdf]

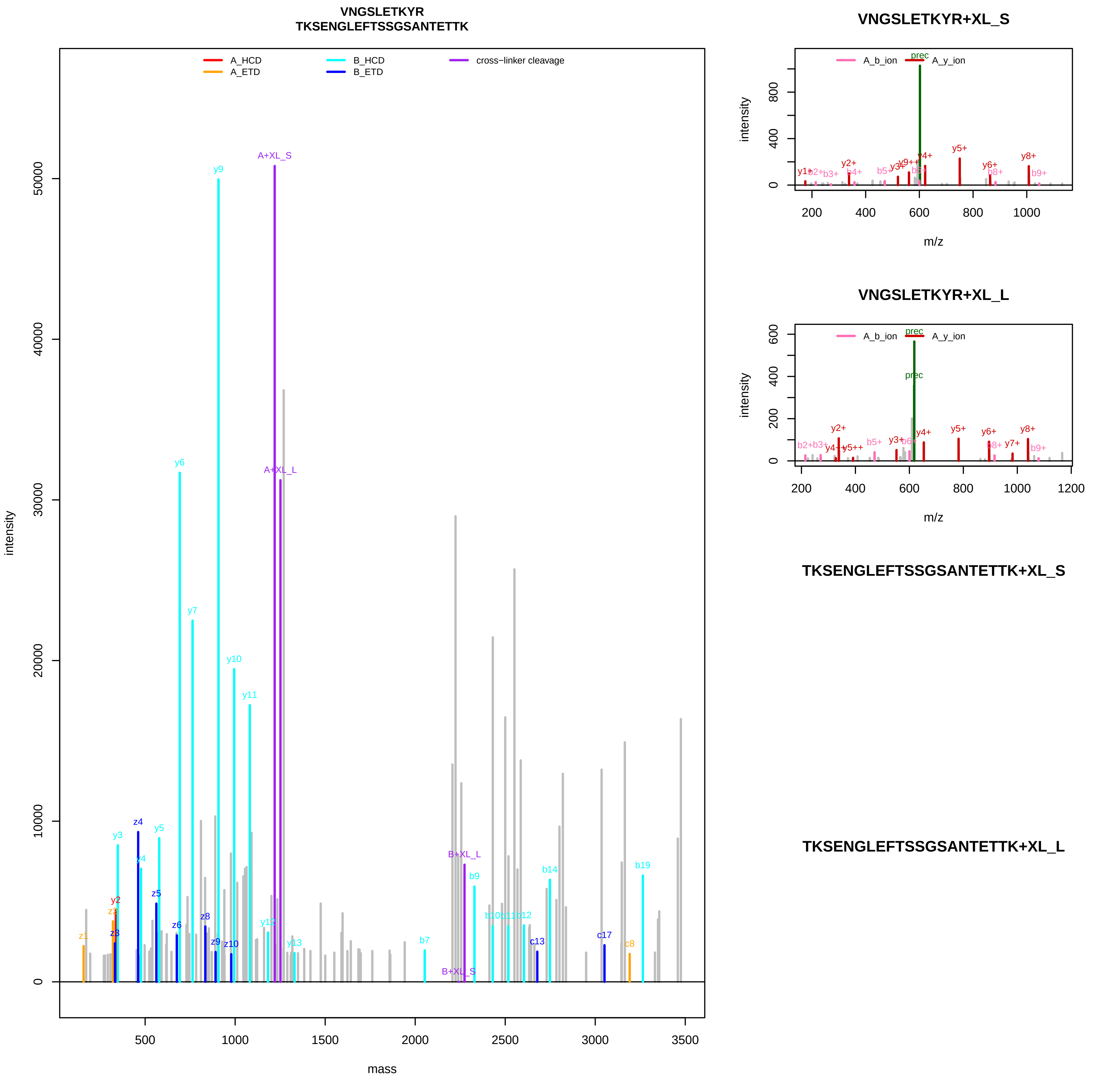
VNGSLETKYR
TKSENGLEFTSSGSANTETTK
A_HCD
A_ETD
B_HCD
B_ETD
cross−linker cleavage
intensity
mass
A+XL_S
A+XL_L
B+XL_L
B+XL_S
y2 y3 y4 y5 y6 y7 y9 y10 y11 y12 y13
z1 z3 z4 z5 z6 z8 z9 z10
b7 b9 b10 b11 b12 b14 b19
c8 c13 c17
VNGSLETKYR+XL_S
A_b_ion
A_y_ion
prec
intensity
m/z
y1+ b2+ b3+ y2+ b4+ b5+ y3+ y9++ b6+ y4+ y5+ y6+ b8+ y8+ b9+
VNGSLETKYR+XL_L
A_b_ion
A_y_ion
prec
intensity
m/z
b2+ b3+ y2+ y4++ y5++ b5+ y3+ b6+ y4+ y5+ y6+ b8+ y7+ y8+ b9+
TKSENGLEFTSSGSANTETTK+XL_S
TKSENGLEFTSSGSANTETTK+XL_L
